# Complete Genome Sequence of Mycoplasma flocculare Strain Ms42^T^ (ATCC 27399^T^)

**DOI:** 10.1128/genomeA.00124-15

**Published:** 2015-03-12

**Authors:** Michael J. Calcutt, Mark F. Foecking, Manijeh B. Heidari, Mark A. McIntosh

**Affiliations:** a Department of Veterinary Pathobiology, University of Missouri, Columbia, Missouri, USA; b Department of Molecular Microbiology and Immunology, University of Missouri, Columbia, Missouri, USA

## Abstract

Mycoplasma flocculare is a commensal or low-virulence pathogen of swine. The complete 778,866-bp genome sequence of M. flocculare strain Ms42^T^ has been determined, enabling further comparison to genomes of the closely related pathogen Mycoplasma hyopneumoniae. The absence of the *p97* and *glpD* genes may contribute to the attenuated virulence of M. flocculare.

## GENOME ANNOUNCEMENT

Mycoplasma flocculare is generally considered to be a commensal colonizer of the porcine nasopharynx but an opportunist pneumonic pathogen in coinfections with Mycoplasma hyopneumoniae (1). M. hyopneumoniae is the agent of swine enzootic pneumonia, a disease with high morbidity that significantly impacts swine production (2). Based on 16S rRNA phylogeny, M. flocculare and M. hyopneumoniae are each other’s closest relatives (3), prompting comparative analysis of representative genomes of these taxa (4). A draft genome sequence of M. flocculare ATCC 27716 (15 contigs; 763,948 bp) has been determined and extensively analyzed by comparative genomics and transcriptional profiling (4, 5). Presented herein is the completely assembled genome of the M. flocculare type strain Ms42^T^ (ATCC 27399^T^), the parental strain from which isolate ATCC 27716 was derived by three additional filter cloning steps.

Genomic DNA was prepared from strain Ms42^T^ (obtained from the American Type Culture Collection as ATCC 27399^T^) and sequenced at the National Center for Genomics Research (Santa Fe, NM, USA) using the Pacific Biosciences platform. Reads from two SMRT cells were assembled into a single contig using HGAP version 2 (6) with 574× coverage. An approximately 10.9-kb duplication was verified by PCR and Sanger sequencing. The 778,866-bp genome was auto-annotated using the PGAP pipeline at NCBI, followed by manual curation. The complete genome comprises 629 genes: 563 open reading frames (ORFs), 31 pseudogenes (22 independently confirmed with alternate sequencing chemistries), 30 tRNAs, and 3 rRNA genes with the 5S rRNA gene separated from the 16S-23S rRNA operon. The G+C content is 28.94%.

A comprehensive comparative analysis of the genomes of several swine mycoplasmas has been documented (4). In that study, it was noted that although multiple adherence-related genes of the M. hyopneumoniae P97/P102 families were present in M. flocculare, orthologs encoding the primary cilium adhesin P97 (7, 8) and downstream ORF P102 were absent. The same holds true for parental strain Ms42^T^, indicating that the absence of the P97-P102 locus was not due to inadvertent selection of a deletion during subcloning.

Another feature that may contribute to the differential virulence of M. flocculare and M. hyopneumoniae is the absence of *glpD* (encoding glycerol 3-phosphate oxidase) in M. flocculare. Orthologs of *glpD* have been found in multiple Mycoplasma species, including the six M. hyopneumoniae isolates for which genome sequences are available. The product, GlpD, is responsible for the glycerol-dependent production of H_2_O_2._ This activity has been shown to be cytotoxic to eukaryotic cells when infected by Mycoplasma mycoides subspecies mycoides SC biotype (9), Mycoplasma pneumoniae (10), and Mycoplasma gallisepticum (11), although in the latter study, *glpD* was found to be dispensable for virulence in an infection model. The recent development of genetic tools for M. hyopneumoniae (12) should enable the potential roles of *glpD* and H_2_O_2_ production to be ascertained.

The first completely assembled M. flocculare genome will enable more detailed analyses of genome structure and plasticity among the neurolyticum cluster of Mycoplasma species and provides a complete reference for further postgenomic applications.

### Nucleotide sequence accession number.

This complete genome sequence has been deposited at DDBJ/EMBL/GenBank under the accession number CP007585.

## References

[1] Meyling A, Friis NF 1972 Serological identification of a new porcine mycoplasma species, *M. flocculare*. Acta Vet Scand 13:287–289. 4675802 10.1186/BF03548589 PMC8561508

[2] Opriessnig T, Giménez-Lirola LG, Halbur PG 2011 Polymicrobial respiratory disease in pigs. Anim Health Res Rev 12:133–148. doi:10.1017/S1466252311000120. 22152290

[3] Stemke GW, Laigret F, Grau O, Bové JM 1992 Phylogenetic relationships of three porcine mycoplasmas, *Mycoplasma hyopneumoniae*, *Mycoplasma flocculare*, and *Mycoplasma hyorhinis*, and complete 16S rRNA sequence of *M. flocculare*. Int J Syst Bacteriol 42:220–225. doi:10.1099/00207713-42-2-220. 1374621

[4] Siqueira FM, Thompson CE, Virginio VG, Gonchoroski T, Reolon L, Almeida LG, da Fonsêca MM, de Souza R, Prosdocimi F, Schrank IS, Ferreira HB, de Vasconcelos AT, Zaha A 2013 New insights on the biology of swine respiratory tract mycoplasmas from a comparative genome analysis. BMC Genomics 14:175. doi:10.1186/1471-2164-14-175. 23497205 PMC3610235

[5] Siqueira FM, Gerber AL, Guedes RL, Almeida LG, Schrank IS, Vasconcelos AT, Zaha A 2014 Unravelling the transcriptome profile of the swine respiratory tract mycoplasmas. PLoS One 9:e110327. doi:10.1371/journal.pone.0110327. 25333523 PMC4198240

[6] Chin CS, Alexander DH, Marks P, Klammer AA, Drake J, Heiner C, Clum A, Copeland A, Huddleston J, Eichler EE, Turner SW, Korlach J 2013 Nonhybrid, finished microbial genome assemblies from long-read SMRT sequencing data. Nat Methods 10:563–569. doi:10.1038/nmeth.2474. 23644548

[7] Zhang Q, Young TF, Ross RF 1995 Identification and characterization of a *Mycoplasma hyopneumoniae* adhesin. Infect Immun 63:1013–1019. 7868222 10.1128/iai.63.3.1013-1019.1995 PMC173103

[8] Hsu T, Artiushin S, Minion FC 1997 Cloning and functional analysis of the P97 swine cilium adhesin gene of *Mycoplasma hyopneumoniae*. J Bacteriol 179:1317–1323. 9023217 10.1128/jb.179.4.1317-1323.1997 PMC178831

[9] Pilo P, Vilei EM, Peterhans E, Bonvin-Klotz L, Stoffel MH, Dobbelaere D, Frey J 2005 A metabolic enzyme as a primary virulence factor of *Mycoplasma mycoides* subsp. *mycoides* small colony. J Bacteriol 187:6824–6831. doi:10.1128/JB.187.19.6824-6831.2005. 16166545 PMC1251598

[10] Hames C, Halbedel S, Hoppert M, Frey J, Stülke J 2009 Glycerol metabolism is important for cytotoxicity of *Mycoplasma pneumoniae*. J Bacteriol 191:747–753. doi:10.1128/JB.01103-08. 19028882 PMC2632104

[11] Szczepanek SM, Boccaccio M, Pflaum K, Liao X, Geary SJ 2014 Hydrogen peroxide production from glycerol metabolism is dispensable for virulence of *Mycoplasma gallisepticum* in the tracheas of chickens. Infect Immun 82:4915–4920. doi:10.1128/IAI.02208-14. 25156740 PMC4249280

[12] Maglennon GA, Cook BS, Deeney AS, Bossé JT, Peters SE, Langford PR, Maskell DJ, Tucker AW, Wren BW, Rycroft AN 2013 Transposon mutagenesis in *Mycoplasma hyopneumoniae* using a novel mariner-based system for generating random mutations. Vet Res 44:124. doi:10.1186/1297-9716-44-124. 24359443 PMC4028751

